# Effect of Frozen-Thawed Embryo Transfer on the Metabolism of Children in Early Childhood

**DOI:** 10.3390/jcm12062322

**Published:** 2023-03-16

**Authors:** Ze-Han Dong, Ting Wu, Chen Zhang, Kai-Zhen Su, Yan-Ting Wu, He-Feng Huang

**Affiliations:** 1 The International Peace Maternity and Child Health Hospital, School of Medicine, Shanghai Jiao Tong University, Shanghai 200030, China; 2 Shanghai Key Laboratory of Embryo Original Diseases, Shanghai 200030, China; 3 Institute of Reproduction and Development, Obstetrics and Gynecology Hospital, Fudan University, Shanghai 200011, China; 4 Research Units of Embryo Original Diseases, Chinese Academy of Medical Sciences (No. 2019RU056), Shanghai 200030, China; 5 Key Laboratory of Reproductive Genetics (Ministry of Education), Department of Reproductive Endocrinology, Women’s Hospital, Zhejiang University School of Medicine, Hangzhou 310058, China

**Keywords:** frozen-thawed embryo transfer, metabolomic, metabolite, BMI, SCFAs, bile acids

## Abstract

Background: As a routine procedure in assisted reproductive technology (ART), it is crucial to assess the safety of frozen and thawed embryo transfer (FET). We aimed to investigate the metabolic profile of children conceived through FET in their early childhood. Method: A total of 147 children between the age of 1.5 and 4 years old, conceived through FET or naturally conceived (NC), were recruited. A total of 89 children, 65 in the FET group and 24 in the NC group (matched with the FET group based on children’s BMI) were included in the final statistical analysis of biochemical markers and metabolomics. Results: Children conceived through FET had a lower level of fasting insulin level and HORM-IR and a higher level of fasting glucose and APOE as compared to children naturally conceived. Metabolomics showed that there were 16 small differential metabolites, mainly including amino acids, carnitines, organic acids, butyric, and secondary bile acid between two groups, which enriched in Nitrogen metabolism, Butanoate metabolism, Phenylalanine metabolism, and D-Arginine and D-ornithine metabolism pathways. Conclusion: Although the FET group had a significantly higher level of APOE and fasting glucose, it cannot yet be considered that children in the FET group had an obvious disorder of glucose and lipid metabolism. However, the potentially more active intestinal flora and lower carnitine levels of children in the FET group suggested by metabolomics are worth further exploration.

## 1. Introduction

As the most widely used treatment for infertility, more than 8 million children worldwide have been born through assisted reproductive technology (ART) up to 2018 [1]. Since the first successful frozen-thawed embryo transfer (FET) was reported in 1983, it has become a very important part of assisted reproductive technology (ART) [2]. Even though FET can significantly increase the cumulative pregnancy rate and reduce the risk of multiple gestations and ovarian hyperstimulation syndrome (OHSS) [3], there have been concerns regarding its potential adverse effects. FET has been identified as a potential risk factor for pregnancy-induced hypertension, large for gestational age, and macrosomia [4,5,6,7], all of which might raise the risk of metabolic dysfunction and cardiovascular problems [8,9]. In addition, the exposure of gametes and embryos to the non-physiological environment during the critical preimplantation period may lead to epigenetic disorder of the growth and metabolic systems of the offspring and result in potential long-term health effects.

Animal studies have shown that IVF-ET (in vitro fertilization and embryo transfer) offspring have impaired glucose metabolism, including altered fasting glucose levels and impaired glucose tolerance (IGT) [10,11,12,13]. Recently, we discovered that FET-conceived male mouse offspring had glucose metabolic abnormalities, mainly manifesting insulin resistance [14]. Observations from human studies indicate that body fat composition in IVF children is disturbed, and children conceived by IVF/ICSI (intracytoplasmic sperm injection) have less favorable glucose and cardiovascular metabolic profiles in childhood when compared with naturally conceived children [15,16]. However, human research on the metabolic profile of children conceived through FET is limited. One follow-up study found that children conceived through FET frequently have abnormal lipid metabolism. [17].

Metabolomics, also known as the comprehensive profiling of small molecule metabolites in cells, is the study of the types, quantities, and changes of endogenous metabolites in biological systems, which has undergone a rapid evolution in the past two decades [18]. In the present study, we aimed to investigate the metabolic profile of FET offspring in early childhood at the level of macromolecular metabolites and small molecule changes and explore its potential impact on the metabolism in early childhood, with the hope to conduct early interventions to possible metabolic abnormalities of FET offspring.

## 2. Method

### 2.1. Study Design and Population

This study was a prospective study. From September 2018 to November 2019, 182 children born from FET and 66 naturally conceived children were recruited based on the electronic Case Report Forms (e-CRF) data in the International Peace Maternity and Child Health Hospital (IPMCH). Inclusion criteria were singleton birth children born after 28 gestational weeks and aged 1.5–4 years on the follow-up day. The exclusion criteria were as follows: (1) The mother had a history of severe liver and kidney dysfunction, diabetes, cancer, or autoimmune system disease; (2) One or both of their parents’ BMI was greater than 28 before pregnancy; (3) Children with severe congenital malformations, chromosomal abnormalities, or congenital metabolic diseases (as described in the previous study [19,20]); (4) Children developed from embryo preserved by the conventional slow freezing method.

In total, 118 children from the FET group and 29 from the NC group who consented to provide venous blood were included in biochemical and metabolomic analyses. For a better comparison between the two groups, we matched NC cases with FET cases based on children’s BMI at the ratio of 1:3; in all, 24 children naturally conceived and 65 children born from FET were finally included for statistical analysis.

This study was approved by the hospital’s scientific research ethics committee ((GKLW) 2016-21), and each participant signed an informed consent form for sample and data collection.

### 2.2. Medical History

Sociodemographic characteristics, birth characteristics, and other potential confounding factors were collected by a questionnaire that was administered by specially trained investigators. Specifically, month age = (date of examination—date of birth)/30, gestational week (GW) refers to the exact days of the gestational week (e.g., 39 weeks + 5 days = 39.7 weeks). The diagnosis of GDM (gestational diabetes mellitus) was confirmed if fasting plasma glucose was ≥5.1 mmol/L (≥92 mg/dl), 1-h plasma glucose was ≥10.0 mmol/L (≥180 mg/dl), or 2-h plasma was ≥8.5 mmol/L (≥153 mg/dL). Preterm birth (PTB): gestational week <37 weeks; low birthweight (LB): birthweight < 2500 g; macrosomia: birth weight ≥ 4000 g; birth weight ≤10th percentile was defined as small for gestational age (SGA), and ≥90th percentile was defined as large for gestational age (LGA). As for alcohol consumption, a positive result was defined as having consumed alcohol at least once one year before childbirth. A positive result for smoking was defined as a history of smoking, and a positive result for passive smoking was defined as exposure to second-hand smoking during pregnancy.

### 2.3. IVF Procedures

The ovarian stimulation protocol includes conventional protocols (GnRH-agonist long protocol, short protocol, and GnRH antagonist protocol), modified mild protocol, and individualized combined protocol [21,22,23,24]. The choice of different protocols is based on patients’ age, infertility diagnosis, and ovarian reserve test results. Oocytes were collected 34–36 h after ovulation induction [25] and inseminated using traditional IVF or ICSI. Fertilized oocytes were cultured in a cleavage medium until day 2 or day 3 before being cryopreserved via vitrification [26]. Natural cycles, hormone replacement cycles, and human menopausal gonadotropin (HMG)-stimulated cycles were used to administer endometrial preparation [27]. The embryos were thawed and transferred on a day when the maternal estradiol levels and endometrial thickness were well-prepared.

### 2.4. Biochemical Analysis

The children’s peripheral venous blood was collected after they were fasting (>8 h). Plasma was collected in EDTA tubes after centrifugation at 3000 rpm for 10 min and stored in a −80 °C refrigerator. Fasting blood glucose (FBG) was measured using a glucometer ((ACCU-CHEK (Roche) glucose meter). Plasma concentrations of total cholesterol (CHOD-POD method), triglyceride (GPO-PAP method), HDL-cholesterol (direct method), and LDL-cholesterol (direct method) were measured by TBA 120 FR chemistry analyzer (Toshiba Co., Tokyo, Japan) [28]. The original formula was used to calculate the homeostasis model assessment (HOMA) as a marker of insulin resistance [29].

### 2.5. Metabolomic Analysis

#### 2.5.1. Sample Preparation

All targeted metabolite standards were obtained from Sigma-Aldrich (St. Louis, MO, USA), Steraloids Inc. (Newport, RI, USA), and TRC Chemicals (Toronto, ON, Canada). All standards were precisely weighed and prepared in water, methanol, sodium hydroxide solution, or hydrochloric acid solution to yield individual stock solutions containing 5.0 mg/mL. An appropriate amount of each stock solution was mixed to create stock calibration solutions. To reduce sample degradation, samples were thawed on ice, 25 μL of plasma was added to a 96-well plate, and the plate was transferred to the workstation (Biomek 4000, Beckman Coulter, Inc., Brea, CA, USA). Each sample received 100 μL of ice methanol with partial internal standards and was vortexed vigorously for 5 min.

The plate was returned to the workstation after centrifugation at 4000 g for 30 min (Allegra X-15R, Beckman Coulter, Inc., Indianapolis, IN, USA). Then, 30 μL of supernatant was transferred to a clean 96-well plate, and each well-received 20 μL of freshly prepared derivative reagents. 350 μL of ice-cold 50% methanol solution was added to dilute the sample after it had been sealed and derivatized at 30 °C for 60 min. The plate was then stored at −20 °C for 20 min before being centrifuged at 4 °C for 30 min. 135 μL of supernatant was transferred to a new 96-well plate, each with 15μL internal standards. The left wells were treated with serial dilutions of derivatized stock standards.

#### 2.5.2. Data Analysis and Processing

Metabolomic analysis was performed against 210 standard metabolites using an ultra-performance liquid chromatography coupled to tandem mass spectrometry (UPLC-MS/MS) system (ACQUITY UPLC-XEVO TQ-S, Waters Corp., Milford, MA, USA); a similar method was prescribed in previous studies [30,31]. The following are brief descriptions of the optimized instrument settings: Temperatures for the sample manager and column were set to 10 °C and 40 °C, respectively. The injection volume was set at 5 μL, with a flow rate of 0.4 mL/min. The mobile phases were 0.1% formic acid (A) and acetonitrile (B). The gradient conditions were set as follows: 0–1 min, 5% B; 1–12 min, 5% → 80% B; 12–15 min, 80–95% B; 15–16 min, 100% B; 18–18.1 min, 100% → 5% B, 18.1–20 min, 5% B. The following instrument parameters were chosen for the mass spectrometer: voltage,1.5 Kv (ESI+)2.0(ESI−), source temperature, 150 °C; desolvation temperature, 550 °C; desolvation gas flow, 1000 L/h. The raw data files generated by UPLC-MS/MS were processed using Quan MET software (v2.0, Metabo Profile, Shanghai, China) to perform peak integration, calibration, and quantification of each metabolite.

### 2.6. Statistical Analysis

The propensity score matching method (PSM) was adopted to match the NC cases for the FET cases. Statistical data analysis was conducted using SPSS (IBM, Armonk, NY, USA). Continuous variables with normal distribution were presented as mean ± standard deviation (SD), while continuous variables with nonnormal distribution were presented as median (first quartile, third quartile); the differences in the continuous variables between the two groups were tested using the *t*-test or Mann–Whitney U test. Noncontinuous data were presented as a percentage, and differences were detected using the Pearson χ2 test or Fisher’s exact test. A *p*-value less than 0.05 was deemed significant. Based on Xplore MET’s one-stop analysis software platform (including embedded statistical R software (3.2.1) code and a link to the KEGG database) for metabolomic data analysis, interpretation, and visual mapping. Orthogonal projection to latent structure discriminant analysis (OPLS-DA) was used in multivariate statistical analysis. A permutation test (1000 times) was performed for the statistical validation of the OPLS-DA model. Univariate analysis was also performed (*t*-test or Mann–Whitney U Test) to determine the differential metabolites between the two groups. The involved pathways based on the differential metabolites were identified using the KEGG-has library (http://www.genome.jp/kegg-bin/showpathway, accessed on 6 February 2023).

## 3. Results

### 3.1. Baseline Characteristics

The comparison of baseline characteristics between the FET group and the NC group was summarized in Table 1. The median age of children in the NC group on the follow-up day was higher than children in the FET group by about four months. Other characteristics, including maternal and paternal factors, were comparable between the two groups.

### 3.2. Biochemical Profile

The biochemical profile of the FET group compared with the NC group is presented in Table 2. Children in the FET group have a significantly higher concentration of APOE than in the NC group. As for glucose metabolism, the fasting insulin level and HOMA-IR index were significantly decreased, while the fasting glucose level significantly increased in the FET group. No significant difference was observed in other biochemicals between the two groups.

### 3.3. Metabolomic Profile

**Figure 1:**
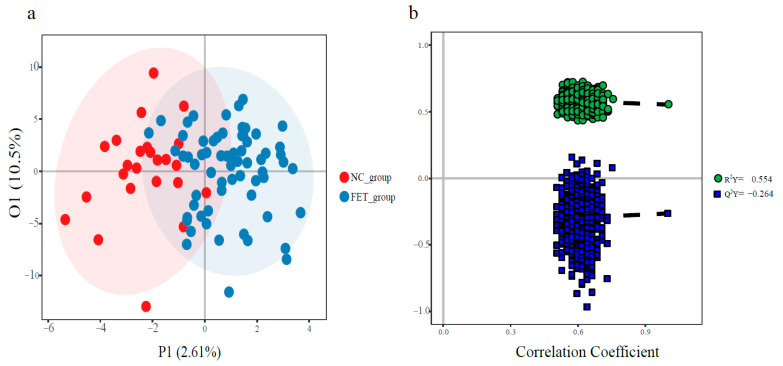
OPLS-DA score plot and permutation test of the OPLS-DA model (**a**) OPLS-DA score plot, each point represents a metabolic profile of a sample; (**b**) permutation test of the model.

**Figure 2:**
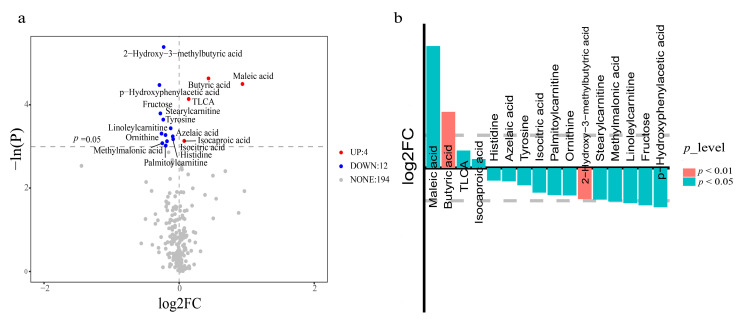
Metabolite profiles of two groups are visualized in (red, upregulated in the FET group, blue, downregulated in the FET group) (**a**) and bar plot (the criteria is log2fc>1, the upper part, upregulated in the FET group, the lower part, downregulated in the FET group) (**b**).

**Figure 3:**
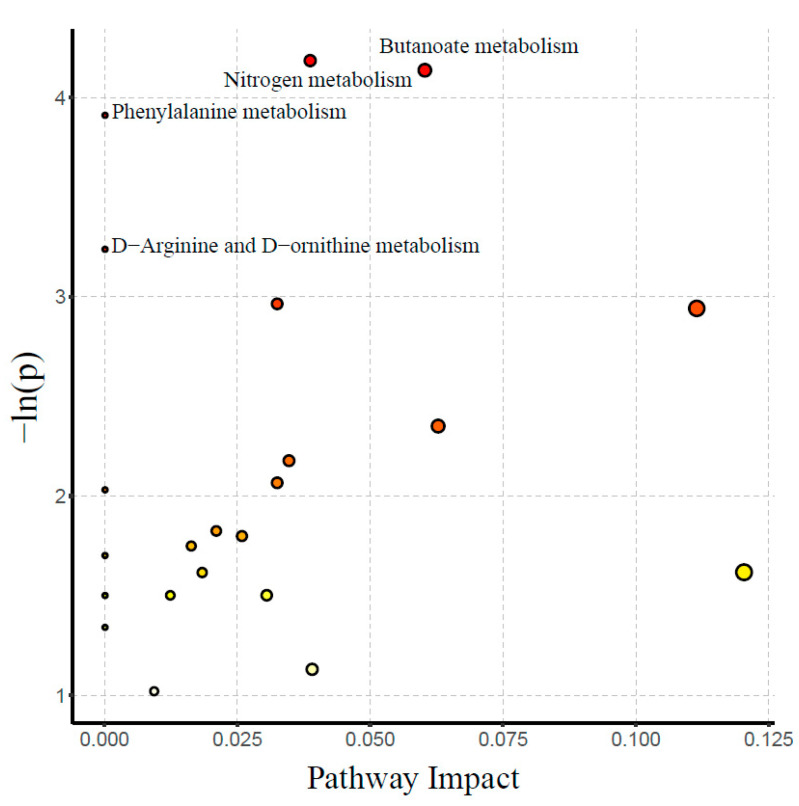
Enriched metabolic pathways based on the screened differential metabolites between the two groups. The color of the point represents the *p*-value. The redder the point is, the more significant the enrichment is. The size of the point represents the number of enriched differential metabolites. Significantly enriched pathways with a *p*-value < 0.05 was highlighted.

In total, 210 small molecule metabolites (µmol/L) from three metabolic pathways, including glucose, amino acid, and lipid metabolism, were detected in plasma; details of category and KEGG number are presented in Table S1. The stacked bars of the relative abundance of various metabolite types in each sample and the Z-score heatmap are shown in Figures S1 and S2, respectively. The results of OPLS-DA (orthogonal partial least-squares discrimination analysis) score plots and the permutation test are presented in Figure 1. However, OPLS-DA failed to completely separate the two groups of small molecule metabolite. Based on univariate analysis, 16 differential metabolites were screened out (Figure 2), with Maleic acid, TLCA (Taurolithocholic acid), and two fatty acids named Butyric acid and Isocaproic acid increased in the FET group. The higher metabolites in the NC group as compared to the FET group were three kinds of amino acid (Histidine, Tyrosine, Ornithine) and organic acid (Azelaic acid, Isocitric acid, 2-Hydroxy-3-methyl-butyric acid, Methylmalonic acid), three kinds of Carnitines (Palmitoyl carnitine, Stearyl carnitine, Linoleyl carnitine), Fructose, and p−Hydroxy phenylacetic acid (Benzenoids). See the box plot (Figure S3) for a more intuitive comparison. According to the Kyoto Encyclopedia of Genes and Genomes (KEGG) database, enriched metabolic pathways based on differential metabolites from two groups were displayed in the bubble plot (Figure 3). The affected pathways were Nitrogen metabolism, Butanoate metabolism, Phenylalanine metabolism, and D-Arginine and D-ornithine metabolism pathways.

## 4. Discussion

### 4.1. Glycolipid Metabolism

Glucose homeostasis plays a critical role in sustaining stable growth and metabolic status for an individual. A meta-analysis found that ART offspring had higher fasting insulin levels but no significant difference in fasting glucose or HOMA-IR when compared to non-ART offspring [32]. A human study demonstrated that children conceived by ART have significantly higher fasting blood glucose and serum insulin levels than children conceived naturally [16]. Our previous animal study showed the decreased insulin tolerance of FET-conceived male offspring, with higher HOMA-IR index and higher serum insulin level post glucose injected than the mouse conceived of natural conception. In addition, mice offspring in both the IVF-chow and FET-chow groups had higher serum TG, LDL, and lower HDL levels than those in the NC-chow group, indicating dyslipidemia [14]. In the present study, the FET group has a decreased level of fasting insulin and HOMA-IR but a higher level of fasting glucose and APOE.

A large number of previous adult-based studies show that the acceleration of lipid decomposition, the entry of total free fatty acid (FFA) into the blood, and the promotion of inflammation are potential intermediate mechanisms of the interaction between obesity and insulin resistance. Adults with obesity and insulin resistance have an accumulation of total FFA, particularly saturated fatty acids (high-risk factor for type 2 diabetes). It is also accompanied by extensive fatty acid oxidation defects, with the products of incomplete fatty acid oxidation accumulating in the circulation. However, there were no significant differences in total FFA (free fatty acids) or SFA (saturated fatty acids) concentrations between the two groups, according to our findings (Table S2). However, the FET group has a significantly higher UFA and PUFA.

Therefore, in the present study, we did not observe significant metabolic characteristics related to glucose and lipid metabolism disorder in the FET group. At the same time, metabolomics showed a decrease in three kinds of carnitines in the FET group. Carnitine is a low-molecular-weight compound that plays a specific role in the mitochondrial oxidation of long-chain fatty acids. Low carnitine availability was suggested to contribute to metabolic inflexibility and impaired glucose tolerance, and carnitine supplementation improves the formation of acetyl-carnitine and rescues metabolic flexibility in IGT subjects [33].

At the same time, a study investigated the plasma profile of subjects with nonalcoholic fatty liver disease (NAFLD), steatosis, and steatohepatitis (NASH) showed higher concentrations of free carnitine, butyryl carnitine, and methyl butyryl carnitine in NASH.

Evidence obtained from animal studies revealed that embryonic exposure to culture components affected the body mass and adiposity of adult offspring in mice [34]. A human study demonstrated that children born after IVF have exaggerated weight gain in late infancy and that such catch-up growth appeared to correlate with the fetal growth pattern itself, regardless of birth weight [35].

Therefore, it cannot be ruled out that children conceived through FET have decreased insulin secretion from beta cells in the pancreatic islet, which results in increased fasting glucose levels, and the changes in lipid metabolism may appear in later childhood period. What is more, it should pay attention to the change in carnitine level in FET offspring and its potential relationship with glycolipid metabolism.

### 4.2. Amino Acid Metabolism

The association between BCAAs, aromatic amino acids, with insulin resistance and type 2 diabetes has been well-established during the past decades [36]. A systematic review evaluating potential metabolite markers of prediabetes and type 2 diabetes suggested that several blood amino acids were associated with the risk of developing type 2 diabetes, including isoleucine, leucine, valine, tyrosine, and phenylalanine [37]. In the children’s study, amino acids linked to insulin resistance, obesity, and impaired glucose tolerance included branched-chain amino acids (BCCAs), phenylalanine (aromatic amino acid), aspartic acid, arginine, histidine, sarcosine, with abnormally high levels of BCCAs being potentially important risk biomarkers [38,39,40,41].

In the present study, no differences were observed in BCAAs between the FET group and the NC group. However, histidine, tyrosine, and ornithine were significantly decreased in the FET group, besides, Phenylalanine metabolism and D-Arginine and D-ornithine metabolism were affected in the FET group as compared to the NC group. Phenylalanine and tyrosine belong to aromatic amino acids, which are essential amino acids for the human body and are mainly produced by intestinal bacteria, especially Escherichia coli. Most phenylalanine is oxidized to tyrosine by phenylalanine hydroxylase in the body. Both are involved in synthesizing essential neurotransmitters and hormones and in the body’s metabolism of glucose and fat body. Histidine is a non-essential amino acid for adults but an essential amino acid for children. Ornithine is mainly involved in the urea cycle and plays an important role in the excretion of nitrogen in the body.

Therefore, the tyrosine production and urea cycle may decrease in children of the FET group. Still, there were no significant changes in amino acid metabolism related to insulin resistance or type 2 diabetes.

### 4.3. Bile Acids (BAs) and Short-Chain Fatty Acids (SFACs) Metabolism

Hepatocytes take cholesterol as raw material and synthesize primary BAs through multiple steps. The primary BAs can combine with glycine, taurine, and other substances to form conjugated Bas, including glycol cholic acid, taurocholic acid, glycol chenodeoxycholic acid, and taurochenodeoxycholic acid. After the BAs are discharged into the intestinal cavity, the conjugated primary BAs are hydrolyzed by bacteria in the ileum and upper colon to free primary BAs, which then undergoes 7-position-dehydroxylation to form secondary BAs- bile acid is converted into deoxycholic acid, and chenodeoxycholic acid is converted into lithocholic acid.

More than 95% of various BAs discharged into the intestine will be reabsorbed. The reabsorbed BAs enter the liver through the portal vein, and the liver cells re-uptake it and convert it into conjugated BAs, which are discharged into the intestine again, thus forming the enterohepatic circulation, which reuses the limited BAs and promotes digestion and absorption of lipids.

SCFAs are primarily produced by the gut microbiota through the fermentation of dietary fiber or carbohydrates [42]; amino acid fermentation also contributes to SCFAs [43]. Unlike the acetate production pathway, which is widely distributed among bacterial groups [43], butyrate production depends on a surprisingly small number of organisms, dominated by *Faecalibacterium prausnitzii*, *Eubacterium rectale*, *Eubacterium hallii*, and *R. bromii* [44]. It is known that SCFAs, particularly butyrate, play a significant role in maintaining the colonic epithelium and act as a preferred fuel of colonocytes.

The increased conjugated secondary BAs (Taurolithocholic acid), butyrate, and the affected butanoate metabolism pathway in the FET group may implicate the more active gut microbiota of children born from FET. In addition, the change in the nitrogen metabolism pathway may be related to the degradation of amino acids by intestinal flora, which can produce SFCA and NH4^+^. The above-mentioned decrease in tyrosine of the FET group may be the result of imbalanced degradation and synthesis of amino acids by intestinal flora. Considering the potential regulatory role of SCFAs in glucose homeostasis, we may speculate that the more active enterohepatic circulation and increased butyrate can promote lipid and glucose metabolism, but several studies indicate the opposite.

A study involving 40 individuals with self-reported diabetes and 60 controls indicates that patients with diabetes exhibit a higher rate of conversion of primary and secondary bile acids by the gut microflora [45]. The study mentioned above showed markedly higher levels of glycocholate, taurocholate, and glycochenodeoxycholate in subjects with NAFLD [46]. Some other studies have reported that intestinal SCFAs concentrations were significantly increased in obese individuals [47,48,49], and high levels of SCFAs may be caused by an imbalance of *phyla Firmicutes* and *Bacteroidetes.* A study based on adolescents suggested that plasma SCFA concentrations were positively related to *phyla Firmicutes/Bacteroidetes*, body mass index, and visceral fat [48]. A high-quality study also revealed that individuals with dysbiosis associated with high fecal SCFAs are prone to increase intestinal permeability, obesity, and cardiovascular disease [50]. Thus, it is interesting to further explore the intestinal flora and BA pool composition of children born from FET.

To summarize, our research found that children conceived through FET have a different metabolite profile as compared to children conceived naturally. Although the FET group has a significantly higher level of APOE and fasting glucose, it cannot yet be considered that children in the FET group have an obvious disorder of glucose and lipid metabolism. However, the potentially more active intestinal flora and lower carnitine levels of children in the FET group suggested by metabolomics are worth further exploration.

To our knowledge, we demonstrated the metabolic profile of children born after FET in early childhood for the first time. However, some limitations should be considered when interpreting our study. First, the sample size of the present study, particularly the NC group, is small. A further study with a larger sample size and a longer follow-up period will be required to observe the long-term metabolic profile of FET offspring. Second, the present statistical method of metabolomics cannot exclude other confounding factors which could influence children’s metabolism, such as dietary patterns and nutrition, to which the parents of the FET group may pay more attention. However, we balanced the children’s BMI between the two groups, which may reduce this impact to some extent. Third, blood tested for metabolomics comes from only one vein of the children, which may not fully reflect their metabolism profile. Further research is needed to explore the intestinal flora and fecal metabolomics of FET offspring.

## 5. Conclusions

Children conceived through FET have a lower level of fasting insulin level, HORM-IR, and a higher level of fasting glucose and APOE. There are 16 small differential metabolites, mainly including amino acids, carnitines, organic acids, butyric, and secondary bile acid, between two groups, which are enriched in four metabolism pathways. The long-term metabolism, intestinal flora, and fecal metabolomics of FET offspring need further exploration.

## Figures and Tables

**Table 1:** Baseline characteristics of singleton children and their parents in the groups.

Children Factors	NC Group (n = 24)	FET Group (n = 65)	*p* Value
Age at follow-up (months)	37.20 (32.05–41.30)	33.50 (26.30–35.30)	0.002 **
Gender			0.651
Boy	12 (50.00%)	29 (44.60%)	
Girl	12 (50.00%)	36 (55.40%)	
BMI (kg/m^2^)	15.40 (14.83–16.15)	15.90 (15.10–16.70)	0.377
Gestational week	38.85 (38.20–39.55)	39.00 (38.00–39.00)	0.303
PTB (<37 weeks)	0 (0.0%)	3 (4.6%)	0.683
Birthweight (g)	3395.00 (3200.00–3586.25)	3460.00 (3000.00–3710.00)	0.757
LBW (<2500 g)	6 (7.2%)	0 (0.0%)	
Macrosomia (>4000 g)	1 (4.2%)	7 (10.8%)	0.583
SGA	0 (0.0%)	6 (9.2%)	0.287
LGA	1 (4.2%)	10 (15.4%)	0.287
NICU	0 (0.0%)	0 (0.0%)	0.379
Breastfeedingtotal duration (months)	6.00 (6.00–11.50)	8.00 (6.00–12.00)	0.703
Maternal factors			
Ovum age (years)	29.50 (27.00–34.00)	32.00 (28.00–35.00)	0.256
Pregnant age (years)	29.50 (27.00–34.00)	32.00 (28.00–35.00)	0.179
BMI (kg/m^2^)	19.95 (18.40–23.35)	19.90 (19.90–21.00)	0.875
Type of delivery			
Cesarean section	7 (29.20%)	21 (32.30%)	0.777
Vaginal	17 (70.80%)	44 (67.70%)	
Smoking	0 (0.0%)	2 (3.1%)	0.949
Passive smoking	10 (41.7%)	29 (45.3%)	0.759
Drinking	8 (33.33%)	9 (13.8%)	0.076
GDM	11 (13.30%)	1 (3.40%)	
Prenatal factors			
Sperm age	31.00 (28.00–33.00)	33.00 (30.00–36.50)	0.077
BMI (kg/m^2^)	24.20 (21.80–26.55)	23.90 (21.95–24.85)	0.694
Smoking	5 (20.8%)	20 (30.8%)	0.355

Abbreviations: NC: natural conception; FET: frozen embryo transfer; LBW: low birth weight; BMI: body mass index; LGA: large for gestational age; SGA: small for gestational age; NICU: neonatal intensive care unit; PTB: preterm birth (gestational week <37 weeks); ** *p* < 0.01.

**Table 2:** Comparison of plasma biochemical markers between the FET group and NC group.

Biochemical Markers	NC Group	FET Group	*p* Value
Fasting blood glucose	5.00 (4.80–5.20)	5.20 (4.90–5.40)	0.049 *
TC (mmol/L)	4.42 (3.88–5.01)	4.39 (3.83–4.98)	0.805
TG (mmol/L)	0.67 (0.54–0.85)	0.70 (0.57–0.87)	0.718
HDL (mmol/L)	1.35 (1.12–1.47)	1.15 (1.36–1.58)	0.372
LDL (mmol/L)	2.25 (1.91–2.99)	1.99 (2.26–2.94)	0.894
APOE (g/L)	179.80 (118.88–235.66)	263.93 (180.95–387.52)	0.007 **
Insulin (IU/L)	4.02 (3.50–6.34)	3.32 (3.00–4.02)	0.002 **
HOMA-IR	0.90 (0.77–1.59)	0.78 (0.68–0.94)	0.018 *
Leptin (pg/mL)	207.58 (108.44–257.65)	229.20 (129.13–322.98)	0.305
FT3 (pmol/L)	4.44 (4.02–6.45)	4.88 (4.27–6.65)	0.240
FT4 (pmol/L)	15.78 (15.17–16.62)	15.61 (14.30–18.24)	0.631
TSH (mIU/L)	3.49 (4.33–6.45)	4.34 (3.65–4.99)	1.000
CRP (ng/mL)	1017.01 (568.47–2263.48)	1039.35 (455.94.47–4467.87)	0.901

Abbreviations: TC: total cholesterol; TG: triglyceride; HDL: high-density lipoprotein; LDL: low-density lipoprotein; APOE: apolipoprotein E; FT3: free Triiodothyronine; FT4: free Thyroxine; TSH: thyroid-stimulating hormone; CRP: C-reactive protein; * *p* < 0.05, ** *p* < 0.01.

## Data Availability

The data that support the findings of this study are available on request from the corresponding author. The data are not publicly available due to privacy or ethical restrictions.

## Supplementary Materials

The following supporting information can be downloaded at: https://www.mdpi.com/article/10.3390/jcm12062322/s1, Figure S1: Stacked bar of relative abundance of various metabolite types in each sample; Figure S2: Z-score heat-map of various metabolites in each sample of the two groups; Figure S3: Box plot of screened differential metabolites between the two groups; Table S1: Names, classifications, and KEGG database number references of the total 210 small metabolites detected; Table S2: Comparison of plasma fatty acid concentrations in children between two groups.
