# Supplementary material for: Effect of Frozen-Thawed Embryo Transfer on the Metabolism of Children in Early Childhood
Source: J Clin Med. 2023 Mar 16;12(6):2322. doi: 10.3390/jcm12062322 (PMC10057347; doi:10.3390/jcm12062322)
Supplement: Supplementary file 1 [file jcm-12-02322-s001.zip › Supplementrary Figure S3.pdf]

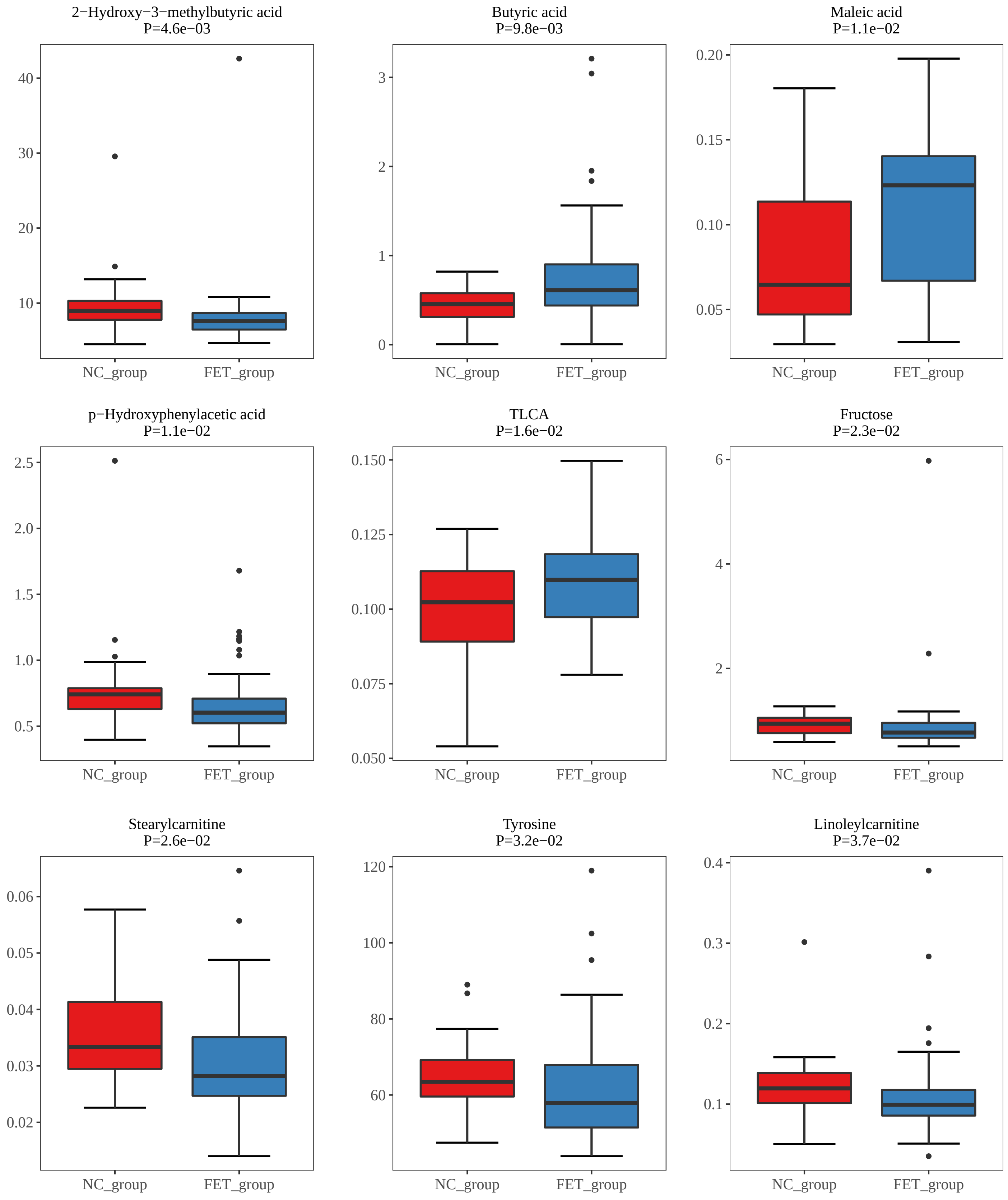
2−Hydroxy−3−methylbutyric acid
P=4.6e−03
40
30
20
10
NC_group
FET_group
Butyric acid
P=9.8e−03
3
2
1
0
NC_group
FET_group
Maleic acid
P=1.1e−02
0.20
0.15
0.10
0.05
NC_group
FET_group
p−Hydroxyphenylacetic acid
P=1.1e−02
2.5
2.0
1.5
1.0
0.5
NC_group
FET_group
TLCA
P=1.6e−02
0.150
0.125
0.100
0.075
0.050
NC_group
FET_group
Fructose
P=2.3e−02
6
4
2
NC_group
FET_group
Stearylcarnitine
P=2.6e−02
0.06
0.05
0.04
0.03
0.02
NC_group
FET_group
Tyrosine
P=3.2e−02
120
100
80
60
NC_group
FET_group
Linoleylcarnitine
P=3.7e−02
0.4
0.3
0.2
0.1
NC_group
FET_group

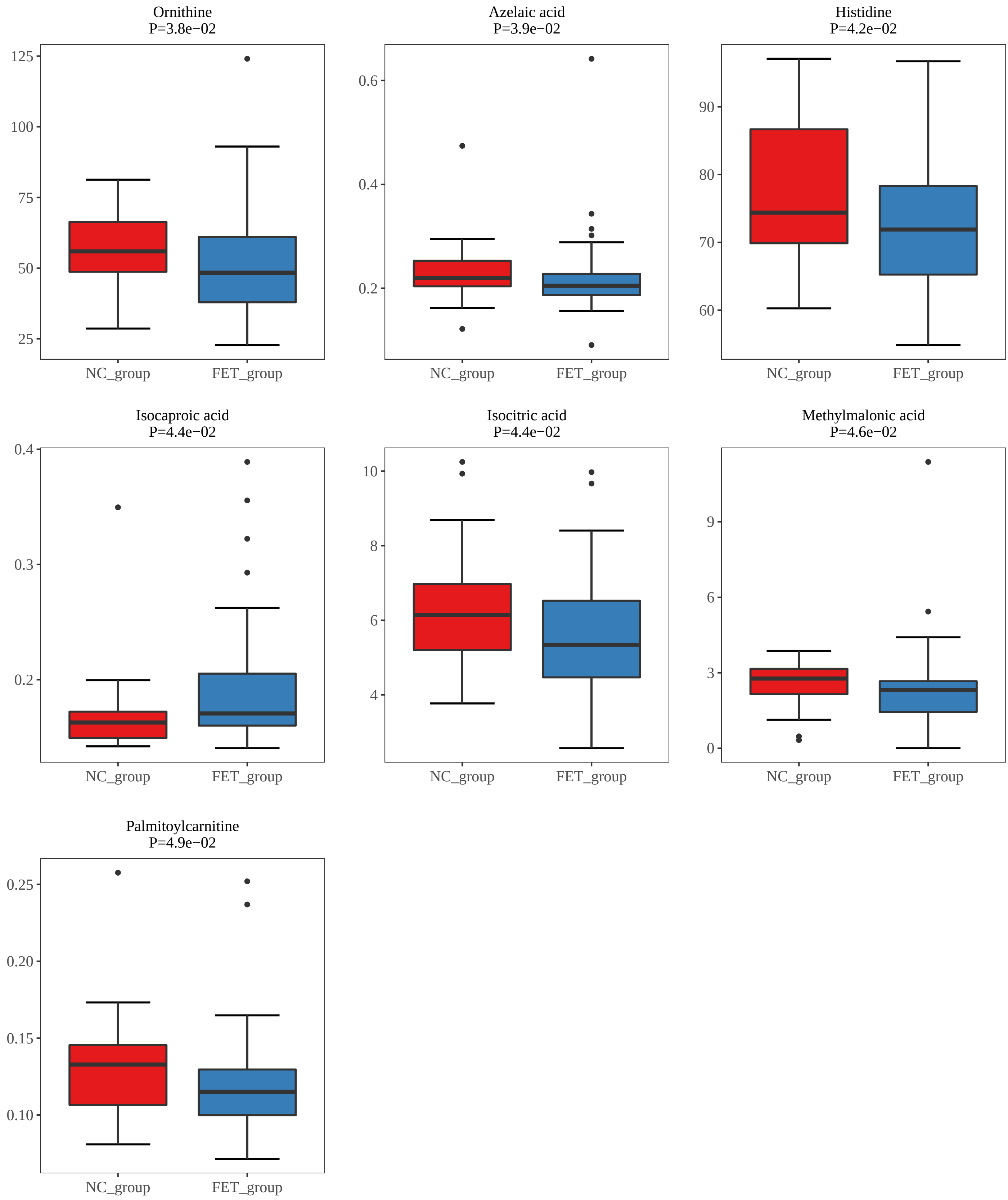
Ornithine
P=3.8e−02
125
100
75
50
25
NC_group
FET_group
Azelaic acid
P=3.9e−02
0.6
0.4
0.2
NC_group
FET_group
Histidine
P=4.2e−02
90
80
70
60
NC_group
FET_group
Isocaproic acid
P=4.4e−02
0.4
0.3
0.2
NC_group
FET_group
Isocitric acid
P=4.4e−02
10
8
6
4
NC_group
FET_group
Methylmalonic acid
P=4.6e−02
9
6
3
0
NC_group
FET_group
Palmitoylcarnitine
P=4.9e−02
0.25
0.20
0.15
0.10
NC_group
FET_group
