# Supplementary figures and images for: Effect of Frozen-Thawed Embryo Transfer on the Metabolism of Children in Early Childhood
Source: J Clin Med. 2023 Mar 16;12(6):2322. doi: 10.3390/jcm12062322 (PMC10057347; doi:10.3390/jcm12062322)

NC\_group

FET\_group

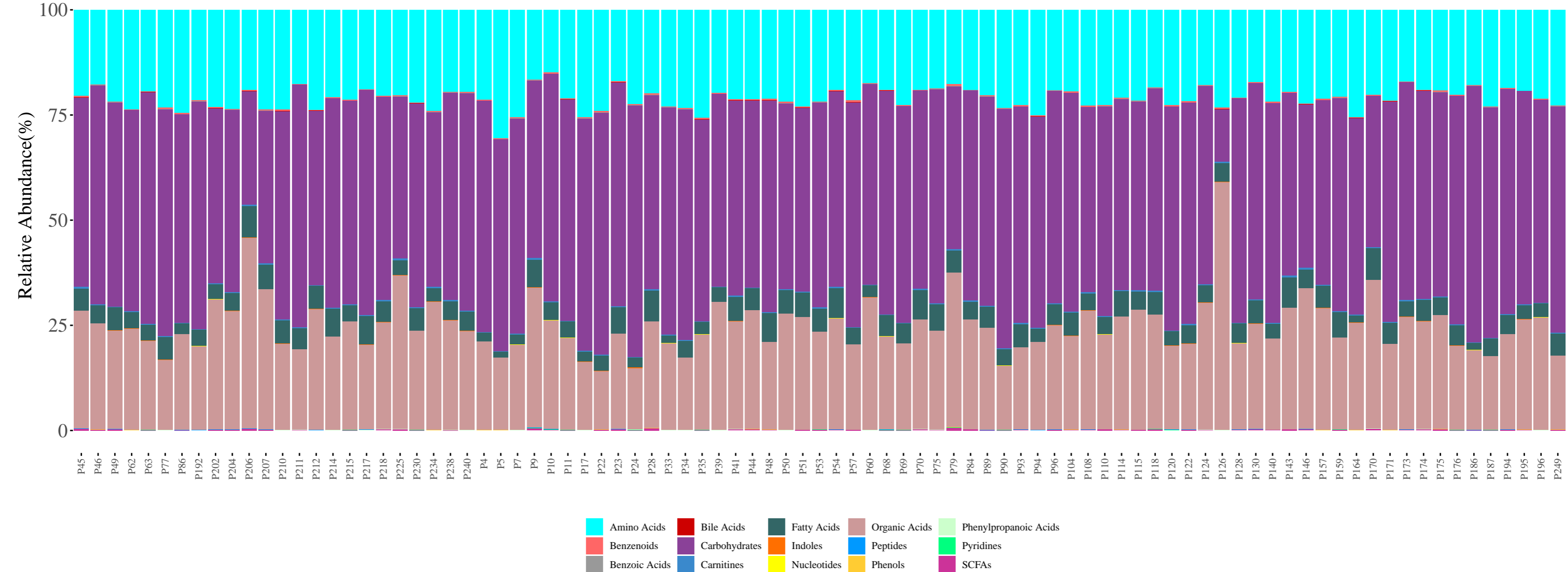

Supplement: Supplementary file 1 [file jcm-12-02322-s001.zip › Supplementary Figure S1.pdf]

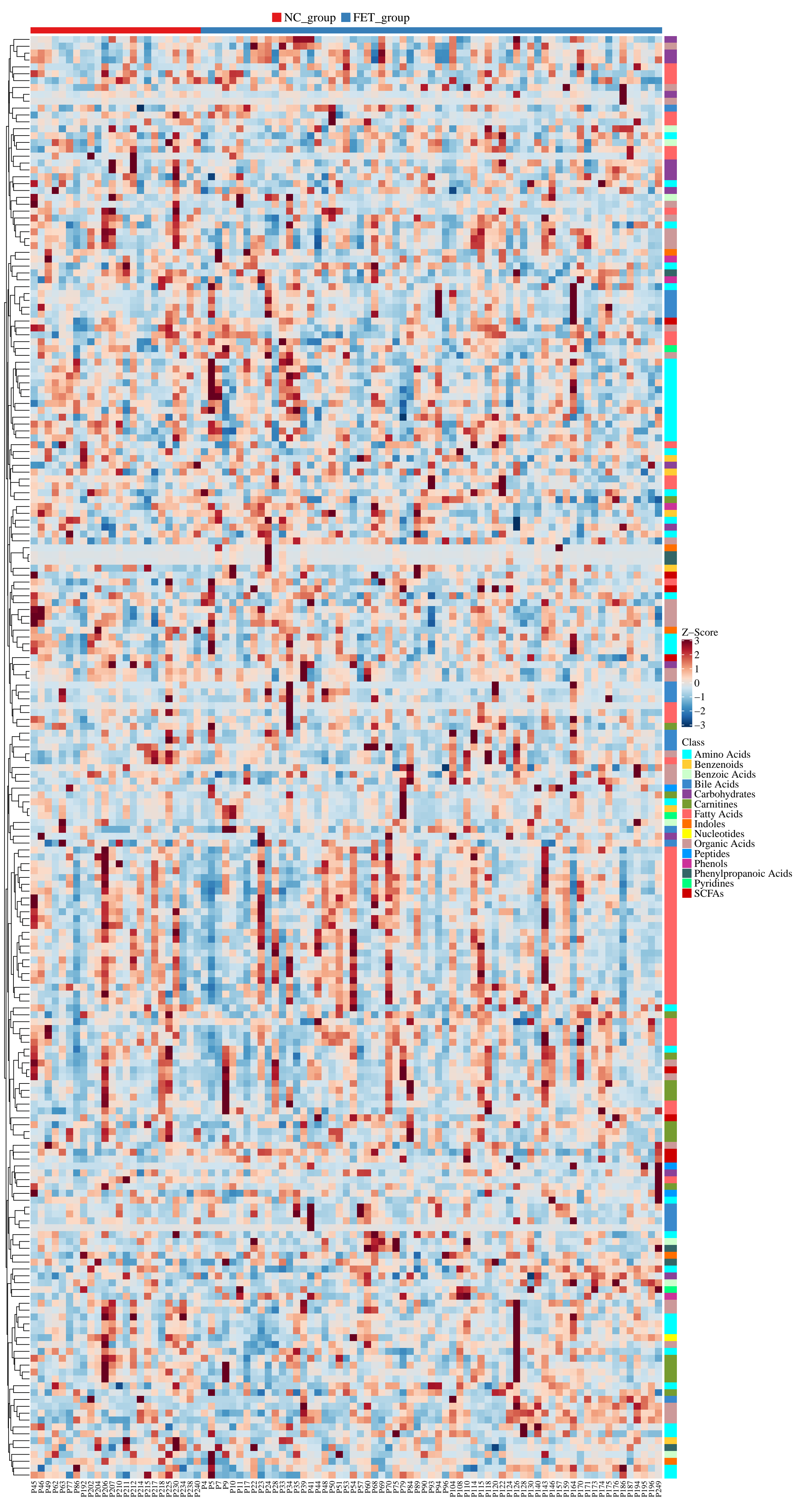

Supplement: Supplementary file 1 [file jcm-12-02322-s001.zip › Supplementray Figrue S2.pdf]
